# Toxic Shock Syndrome Triggered by Thoracic Drain Insertion: A Pediatric Case Report

**DOI:** 10.70352/scrj.cr.25-0226

**Published:** 2025-07-09

**Authors:** Kanako Omata, Mami Ishida, Takahiro Shindo, Mika Nagao, Yoshiyuki Namai

**Affiliations:** Department of Pediatrics, Ohta General Hospital Foundation, Ohta Nishinouchi Hospital, Koriyama, Fukushima, Japan

**Keywords:** toxic shock syndrome, pneumothorax, chest drain

## Abstract

**INTRODUCTION:**

Postoperative Staphylococcus and streptococcal toxic shock syndrome (TSS) are associated with significant morbidity and mortality rates. As a result, surgical awareness is required to recognize and treat TSS appropriately. We report a pediatric case of TSS after thoracentesis for a pneumothorax.

**CASE PRESENTATION:**

A 14-year-old boy was diagnosed with a right pneumothorax and underwent thoracentesis with a trocar catheter. After 2 days, the patient developed a fever, headache, vomiting, and diarrhea. No obvious contamination of the drain puncture wound was observed. He was diagnosed with acute gastroenteritis and received intravenous treatment. On the 4th day after drainage, his blood pressure decreased. Due to suspected septic shock, he was transferred to the intensive care unit and administered antibiotics, immunoglobulin, and a hypertensive agent. His treatment response was good, and his general condition improved relatively quickly. On the 6th day, the patient was discharged from the intensive care unit. Although the air leak from the thoracic drain disappeared on day 3, the drain remained in place until day 8. Blood cultures obtained at the time of septic shock were all negative; however, pleural fluid and thoracic drain tip cultures detected *Staphylococcus aureus*, and the production of TSS toxin-1 and enterotoxin type C was confirmed. Retrospectively, the patient was diagnosed with TSS triggered by the insertion of a thoracic drain. He was discharged from the hospital on day 11. After discharge, he experienced skin desquamation of the axilla and buttocks. The patient also reported diffuse erythematous eczema on day 3 after drainage. He received antimicrobial therapy for 14 days and recovered fully without any complications.

**CONCLUSIONS:**

TSS can occur after simple, routine procedures such as thoracentesis. Symptoms such as fever, rash, vomiting, and diarrhea should raise concern for TSS and prompt further exploration and cultures, even in benign-appearing postoperative wounds.

## Abbreviation

TSS
: toxic shock syndrome

## INTRODUCTION

TSS is a multisystem, life-threatening disease characterized by fever, hypotension, rash, desquamation, and multiple organ failures, such as gastrointestinal symptoms, renal dysfunction, liver failure, thrombocytopenia, and damage to the central nervous system.^1)^ It is predominantly caused by superantigen toxin-producing strains of *Staphylococcus aureus* and *Streptococcus pyogenes* (group A *Streptococcus*).

TSS is generally categorized into menstrual TSS and non-menstrual TSS, depending on the cause of onset. This is due to the reportedly high incidence of TSS in menstruating women using tampons in the 1980s.^2)^ Subsequent changes in tampon manufacturing led to a decrease in the incidence of menstrual TSS; however, the relative rate of TSS has increased among postoperative patients. A French surveillance study in 2008 demonstrated that 65% of cases of staphylococcal TSS were non-menstrual, with a mortality rate of 22%, compared to 0% in menstrual TSS.^3)^

Because TSS is a rapidly progressive and potentially fatal complication of surgery, surgical awareness is required to recognize and treat this condition appropriately. Here, we report a pediatric case of TSS following thoracentesis for a pneumothorax.

## CASE PRESENTATION

**Fig. 1:**
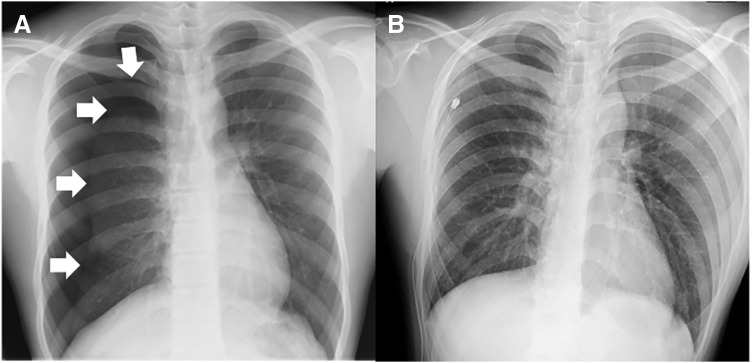
X-rays before (**A**) and after (**B**) insertion of the thoracic drain. (**A**) Chest radiograph showing a right pneumothorax (arrows). (**B**) After insertion of an 18-Fr trocar catheter. The right lung had expanded well.

A 14-year-old boy was referred to our hospital following an episode of chest pain. Upon observation and review of the medical imaging findings, he was diagnosed with a right pneumothorax (**Fig. 1**). He underwent thoracentesis with an 18-Fr trocar catheter, and continuous aspiration at −10 cmH_2_O was initiated. No prophylactic antibiotics were administered. After 2 days, the patient developed a fever of 39°C, headache, vomiting, and diarrhea. No obvious contamination of the drain puncture wound was observed. He was diagnosed with acute gastroenteritis and received intravenous treatment. After 3 days, the patient noticed that a rash had appeared all over his body; however, this was overlooked at that point. On day 4 after insertion of the drain, his blood pressure decreased. Laboratory test results showed a high white blood cell count of 34900/μL and a C-reactive protein level of 30.23 mg/dL. The platelet count was 181000/μL, which was within the normal range. Renal failure was observed, with a serum creatinine level of 1.45 mg/dL. Due to suspected septic shock, he was transferred to the intensive care unit and administered antibiotics (meropenem 3 g/day), immunoglobulin (100 mg/kg), and a hypertensive agent. His treatment response was good, and his general condition improved relatively quickly; however, a remitting fever over 38°C persisted until the day before discharge, when the fever suddenly subsided. On day 6, the patient was discharged from the intensive care unit. Although the air leak from the thoracic drain disappeared on day 3 after drain insertion, the drain remained in place during intensive care and was removed on day 8. Blood cultures obtained at the time of septic shock were all negative; however, pleural fluid and thoracic drain tip cultures detected *Staphylococcus aureus*, and the production of TSS toxin-1 and enterotoxin type C was confirmed.

**Fig. 2:**
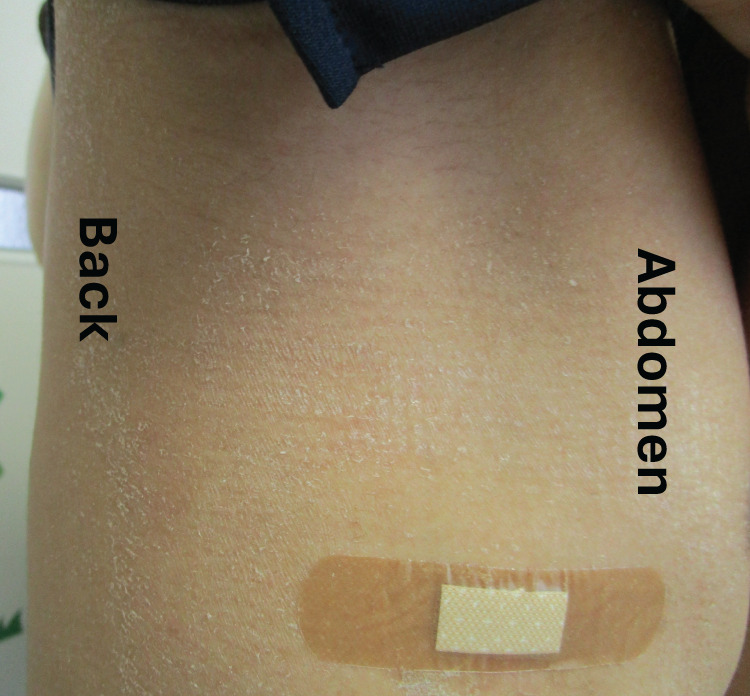
Skin desquamation of the axilla and buttocks on day 10 after insertion of the drain.

The patient was retrospectively diagnosed with TSS triggered by the insertion of a thoracic drain. He was discharged from the hospital on day 11. Following discharge, he experienced skin desquamation of the axillae and buttocks (**Fig. 2**). Furthermore, because the patient reported a sunburn-like rash all over the body on day 3 after drain insertion, we recognized that one of the classic symptoms of TSS had been present. The patient received antimicrobial therapy for 14 days and recovered fully without any complications.

## DISCUSSION

In 1995, a United States report reviewed 39000 patients who underwent surgical procedures and found that postoperative TSS occurred in 0.003% of the patients (12 cases).^4)^ One of these cases was a 26-year-old male who developed TSS after a pneumothorax. In this report, all the patients had a rash, most commonly in a truncal, “sunburn” pattern. The mean time from surgery to symptom onset was 4 days. No correlation was demonstrated between the development of TSS and age, sex, preoperative skin preparation, administration of antibiotics, or duration of the procedure. A systematic review of the literature on postoperative TSS, published in 2020, evaluated 67 reports and 96 patients from 1978 to 2018.^5)^ The mean patient age was 34.1 years. The median number of days to symptom onset or hospital admission was 4 days. Furthermore, 76% of the patients did not experience permanent complications, while 24% did. The mortality rate was 9.38%. The surgeries most commonly preceding TSS involved extensive handling of skin or mucosal surfaces, such as plastic surgery, orthopedic surgery, and otolaryngology. At least 52 out of 96 patients (approximately 54.2%) presented with gastrointestinal symptoms, such as nausea, vomiting, or diarrhea. **Table 1** summarizes the reports since 2020, including our case.^6–17)^ These cases demonstrate that TSS is a potentially fatal complication of surgery and should not be excluded despite young patient age, patient health, or the relative simplicity of a procedure.

**Table 1:** Toxic shock syndrome case reports since 2020 (including our case)

Study	Year	Age	Sex	Surgical procedure	Days to onset	Mortality
Samas et al.^6)^	2020	36	F	Scar revision	21 days	No
Gonzalez and Yuen^7)^	2020	13	M	Abdominal scar revision	2 days	No
Gruttadauria et al.^8)^	2021	37	F	Laparoscopic cystectomy	4 days	No
Wang et al.^9)^	2021	43	M	Canal wall down mastoidectomy and tympanoplasty	12 h	Yes
Kim et al.^10)^	2021	31	F	Breast reconstruction	16 days	No
		55	F	Breast reconstruction	5 days	No
Liu et al.^11)^	2021	23	F	Liposuction and fat transfer	10 days	No
Abuzneid et al.^12)^	2021	9	M	Orchidopexy	1 day	No
Lee et al.^13)^	2021	56	M	Hemorrhoidectomy	2 days	Yes
Aslam et al.^14)^	2021	19	F	Living kidney transplantation	5 days	No
Nakamura et al.^15)^	2022	35	F	Breast reconstruction	7 days	No
Fernandes et al.^16)^	2023	62	M	Open reduction and internal fixation of a wrist fracture	7 days	No
Tahri et al.^17)^	2024	50	F	Breast cancer surgery	5 days	No
Our case	2025	14	M	Chest drain insertion	2 days	No

F, female; M, male

Because TSS progresses rapidly and is a fatal condition, surgical awareness is essential. However, an early diagnosis of TSS is challenging due to the following reasons. First, the signs and symptoms of early postoperative TSS may be subtle. Symptoms such as fever, vomiting, diarrhea, and pain are not unique to TSS and can be mistaken for other conditions. In this case, similar to previous reports, the patient presented with fever, vomiting, and diarrhea. We would have been able to diagnose TSS earlier if we had proactively looked for the skin rash, which would have led us to consider the possibility of TSS instead of assuming it was gastroenteritis. Next, the surgical site in TSS patients often appears unremarkable, lacking the typical signs of infection, such as pus or necrosis. In addition, in cases caused by *Staphylococcus aureus*, blood cultures are commonly negative, making it challenging to identify the source of infection.

Treatment for TSS involves source control, supportive care, and antibiotic treatment. Source control is a principle particularly relevant to postoperative patients, as surgical wounds must be considered a potential source of infection despite the lack of typical signs. Once sepsis is diagnosed, antibiotic treatment should be initiated within 1 h, and the requisite cultures should be performed prior to this. We could diagnose TSS using a wound culture test; therefore, we recommend that wound cultures, in addition to standard blood cultures, be performed if a postoperative patient presents with symptoms suggestive of TSS. The current recommendation for empiric antibiotic treatment of suspected sepsis advocates the use of flucloxacillin and 3rd-generation cephalosporins.^18)^ In settings where the rate of methicillin-resistant *Staphylococcus aureus* is high, the initial coverage should include vancomycin. Clindamycin is used to counteract the systemic effects of toxins after they are released. The adjunctive use of intravenous immunoglobulin to treat TSS is theoretically supported by its anti-inflammatory and immunomodulatory properties.^18)^ In the present study, the patient was diagnosed with a severe infection and received high-volume infusions, antibiotics, and γ-globulin at an early stage, which was effective in improving his overall condition; however, the fever persisted even after his general condition improved. This could have been due to uncontrolled pathogenesis caused by the release of cytokines induced by exotoxins.

## CONCLUSIONS

Similar to previous reports, this case demonstrated that TSS can occur in simple, routine procedures performed by surgeons, such as thoracentesis. Symptoms such as fever, rash, vomiting, and diarrhea should raise concern for TSS and prompt exploration and cultures, even in benign-appearing postoperative wounds.

## ACKNOWLEDGMENTS

None.

## DECLARATIONS

### Funding

None.

### Authors’ contributions

KO wrote the manuscript.

MI, TS, MN, and YN provided expert advice.

All authors read and approved the final version of this manuscript for publication.

### Availability of data and materials

The datasets supporting the conclusions of this article are included within the article.

### Ethics approval and consent to participate

This work does not require ethical considerations or approval.

Informed consent to participate in this study was obtained from the patient.

### Consent for publication

Informed consent was obtained from the patient for publication of this case report.

### Competing interests

The authors declare that they have no competing interests.
